# Distinguishing successive ancient polyploidy levels based on genome-internal syntenic alignment

**DOI:** 10.1186/s12859-019-3202-x

**Published:** 2019-12-17

**Authors:** Yue Zhang, Chunfang Zheng, David Sankoff

**Affiliations:** Department of Mathematics and Statistics, University of Ottawa, 150 Louis Pasteur pvt, Ottawa, K1N 6N5 Canada

**Keywords:** Polyploidy, Whole genome doubling, Whole genome tripling, Gene triples, Plant genomes, Branching process

## Abstract

**Background:**

A basic tool for studying the polyploidization history of a genome, especially in plants, is the distribution of duplicate gene similarities in syntenically aligned regions of a genome. This distribution can usually be decomposed into two or more components identifiable by peaks, or local maxima, each representing a different polyploidization event. The distributions may be generated by means of a discrete time branching process, followed by a sequence divergence model. The branching process, as well as the inference of fractionation rates based on it, requires knowledge of the ploidy level of each event, which cannot be directly inferred from the pair similarity distribution.

**Results:**

For a sequence of two events of unknown ploidy, either tetraploid, giving rise to whole genome doubling (WGD), or hexaploid, giving rise to whole genome tripling (WGT), we base our analysis on triples of similar genes. We calculate the probability of the four triplet types with origins in one or the other event, or both, and impose a mutational model so that the distribution resembles the original data. Using a ML transition point in the similarities between the two events as a discriminator for the hypothesized origin of each similarity, we calculate the predicted number of triplets of each type for each model combining WGT and/or WGD. This yields a predicted profile of triplet types for each model. We compare the observed and predicted triplet profiles for each model to confirm the polyploidization history of durian, poplar and cabbage.

**Conclusions:**

We have developed a way of inferring the ploidy of up to three successive WGD and/or WGT events by estimating the time of origin of each of the similarities in triples of genes. This may be generalized to a larger number of events and to higher ploidies.

## Background

**Fig. 1:**
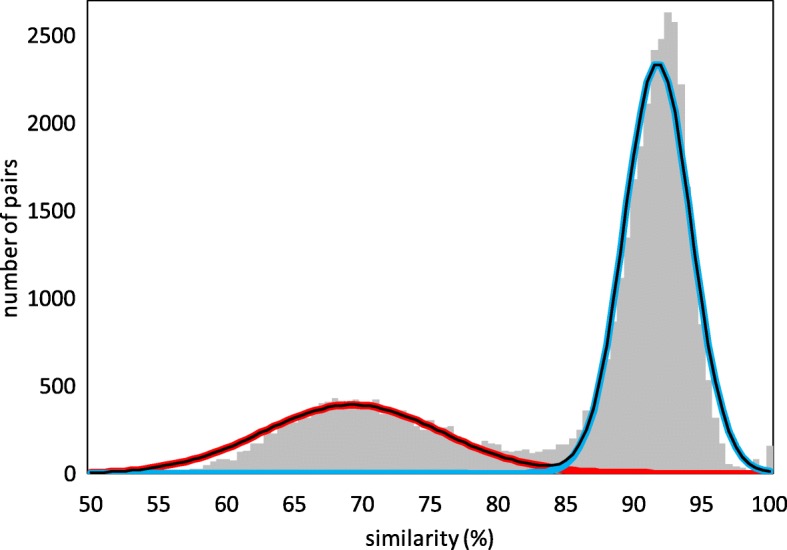
Distribution of gene pair similarities. Pairs in the *Durio zibethinus* (COGE ID 51764) genome after two rounds of whole genome tripling. Discrimination point *H*=85.2*%*. Cut-off for pairs not originating in polyploidization > 98*%*

Given the pervasiveness of whole genome doubling (WGD) and tripling (WGT) in the ancestral lineages of plant species, a widespread feature of plant genome publications is the display of the distribution of duplicate gene identities (or similarities, distances, *K*_*s*_,...). This is illustrated in Fig. 1, which represents the distribution of similarities between syntenically aligned duplicate genes [1, 2] in the durian (*Durio zibethinus*) genome [3]. The two visually distinguishable but overlapping parts of the distribution are the legacy of two successive polyploidization events. The means (*t*_1_ and *t*_2_), variances and proportion of the total sample of each component of the distribution can be estimated by mixtures of models techniques such as EMMIX [4].

These distributions can be explained and generated by a discrete-time branching process model of polyploidization and fractionation (not time-homogeneous), mathematically represented by the product of successive *r*-nomial distributions, the output of one being the input of the next, where the parameters, namely the *r*-nomial probabilities, express resistance to gene loss through fractionation [5–9].

These parameters, which are key to understanding the cycle of polyploidization and fractionation, can be estimated using the information inferred from the distribution of gene pair similarities.

One aspect of the branching process that cannot be inferred from the study of *pairs* only is the ploidy *r* of the various events. Thus, durian is known to have undergone two whole genome triplings [9, 10], the *γ* tripling almost 120 million years ago [11], ancestral to most flowering plants, and a more recent tripling (10-20 Mya) not shared by even closely related species, like cacao. By just looking at the distribution of similarities in Fig. 1 engendered by these events, however, there is no direct way of knowing whether one or both of the two component distributions represent WGD, WGT, or other polyploid events. To resolve this problem, the main point of this presentation, we propose to add *triples* of similar (> 50 %) genes to the study of gene pairs. Our technique, based on the branching process, responds to our concern in previous ad hoc treatments [7, 12] of how to use triples rigorously from a statistical point of view.

In the next section, we summarize the general branching process approach to analyzing the distribution of gene pair similarities. We then focus on four competing two-event models involving WGD and/or WGT. We define four types of gene triplet according to whether the gene pairs within them were created by the first event, the second event, or both. Within each model, we calculate the expected number of triplets of each type. Thus creates an “underlying” profile of triplet distribution to compare to the “observed” profile of triplets in the data. Because of the way the two components of the pair similarity distribution overlap, however, the origin of each triplet in the data is not always obvious. Thus we create a “predicted” profile of triplet distribution by grafting a paralog divergence model onto the branching process, making use of a maximum likelihood dividing point between the two components. We apply this analysis to the genomes of durian, poplar *Populus trichocarpa* [13] and cabbage *Brassica oleracea* [14], each of which has a different sequence of polyploidization events. These histories are captured correctly for the first two, but the results for *B. oleracea* prompt an extension to three-event models, which we carry out, and suggest further work to higher numbers of events.

### The branching process and two-event models

Denote by *m*_*i*_ the total number of individuals (genes)at time *t*_*i*_,*i*=1,…,*n*. Set *m*_1_=1. At time *t*_*i*_, *i*=1,…*n*−1, each of the *m*_*i*_ genes is replaced by *r*_*i*_≥2 progeny, but only *j*≥1 of them survive until time *t*_*i*+1_, with probability $u_{j}^{(i)}$.

Of the total of *m*_*i*_ genes at time *t*_*i*_, let $a_{j}^{(i)}$ be the number for which *j* progeny survive until time *t*_*i*+1_, so that

1 $$ m_{i}=\sum_{j=1}^{r_{i}}{a_{j}^{(i)}},\ \ \ \ \ m_{i+1}=\sum_{j=1}^{r_{i}}j{a_{j}^{(i)}}.  $$

The probability distribution of the evolutionary histories represented by the given $\mathbf {r}=\{r_{i}\}_{i=1}^{n-1}$ and the variable $\mathbf {a}=\left \{a_{j}^{(i)}\right \}_{j=1\dots r_{i}}^{i=1\dots n-1}$ is

2 $$ P(\mathbf{r;a})= \prod_{i=1}^{n-1}\Bigg[\binom{m_{i}}{a_{1}^{(i)},\dots,a_{r_{i}}^{(i)}}\prod_{j=1}^{r_{i}} \left(u_{j}^{(i)}\right)^{a_{j}^{(i)}}\Bigg].  $$

The expected number of genes at time *t*_*n*_ is

3 $$ \mathbf{E}(m_{n})=\sum_{\mathbf{a}}P(\mathbf{r;a}){m_{n}}.  $$

**Fig. 2:**
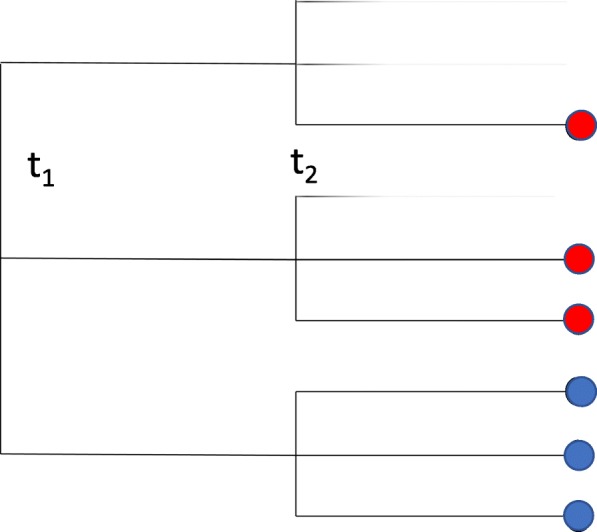
Sample trajectory, starting from a single gene, of branching process based on two whole genome triplings

This is illustrated by the sample trajectory in Fig. 2, in which a WGT at time *t*_1_, with all three progeny surviving – the 3-nomial sample has value 3 – is followed by another independent WGT at time *t*_2_ where the three lineages show one, two or all three offspring surviving, i.e., the independent 3-nomials samples have values 1,2 and 3, respectively. We will study the case of two successive polyploidy events, with *r*_1_ and *r*_2_ taking on values 2 or 3, i.e., WGD or WGT, in all four combinations, i.e., in the set $\mathscr {M}$ of “models”, denoted (3,3),(3,2),(2,3) and (2,2). Because of the limited information that can be inferred about each component of the distribution of similarities, we can only infer the probabilities of samples of value 1, 2, or 3, so that we are limited to 2-nomials and, with some assumptions, 3-nomials, by far the biologically most important cases.

To infer parameters like fractionation rate in the polyploidization history of a genome, based on the distribution of gene pair similarities, we need to know the ploidies *r*_*i*_ of the various events. This motivates us to extend our study from gene pairs only to also include gene triplets.

## Methods

### Triplet probabilities in four models

With no loss of generality, we study triplets of similarities among three genes, rather than the triplets of genes themselves. A triplet is a (multi-)set {*t*_*i*_,*t*_*j*_,*t*_*k*_}, where each of *i*,*j* and *k* may be 1 or 2. Let $\mathscr {T}=\{\{t_{1},t_{1},t_{1}\}, \{t_{1},t_{1},t_{2}\}, \{t_{1},t_{2},t_{2}\},\{t_{2},t_{2},t_{2}\}\}$. We classify each kind of triplet we according to whether each of the three paralogies among the three pairs of gene originates in the first event or the second event. Thus in the branching process illustration in Fig. 2 the blue dots represent genes that form a {*t*_2_,*t*_2_,*t*_2_} triplet and the red dots form a {*t*_1_,*t*_1_,*t*_2_} triplet. The single red dot combines with the three pairs of blue genes to form three additional {*t*_1_,*t*_1_,*t*_2_} triplets. And there are a further nine {*t*_1_,*t*_1_,*t*_2_} triplets in the sample. We can calculate the expected number of triplets of each type by enumerating the triplets of each type in each possible trajectory of the process, and multiplying by the probability of this trajectory from Expression (2). The enumeration within a trajectory is easily done by considering every triple of genes at time *t*_*n*_ and identifying the last common ancestor of each pair. The probabilities of the trajectories have previously been calculated [5–7]. We then sum these results over all trajectories. These are listed in Table 1.

**Table 1:** Formulae for the expected numbers of triplets *W*_*M*_(*Δ*) of each type *Δ*, by branching model *M*

Model $M\in \mathscr {M}$	Tripling-	Tripling-	Doubling-	Doubling-
	tripling	doubling	tripling	doubling
Triplet $\Delta \in \mathscr {T}$	(3,3)	(3,2)	(2,3)	(2,2)
{*t*_1_,*t*_1_,*t*_1_}	*u*^′^(1+2*v*^′^+*v*)^3^	*u*^′^(1+*v*)^3^	-	-
{*t*_1_,*t*_1_,*t*_2_}	2(3*u*^′^+*u*)(3*v*^′^+*v*)	2(3*u*^′^+*u*)	2*u*(3*v*^′^+*v*)	(2*u*)*v*(1+*v*)
	×(1+2*v*^′^+*v*)	×*v*(1+*v*)	×(1+2*v*^′^+*v*)	
{*t*_1_,*t*_2_,*t*_2_}	-	-	-	-
{*t*_2_,*t*_2_,*t*_2_}	(1+2*u*^′^+*u*)*v*^′^	-	(1+*u*)*v*^′^	-

*u*= probability that two progeny survive after the first polyploidization event. *u*^′^= probability that three survive. Similarly *v* and *v*^′^ are the probabilities that two or three progeny survive, respectively, after the second event

This table provides the expected number of triplets *W*_*M*_(*Δ*) of each type $\Delta \in \mathscr {T}$ produced by the branching process for each model $M \in \mathscr {M}$. For a given genome, the four numerical values of *W*_*M*_(·) constitute the *underlying profile* of the model *M*. The underlying profiles for each model based on maximum likelihood values of *u* and *v* are given in the top half of Tables 2, 3 and 4 below. Because of the limited number of parameters that can be inferred from the distribution of similarities, we assume *u*^′^=*u*^2^ and *v*^′^=*v*^2^.

**Table 2:** Durian model predictions before (underlying) and after imposition of mutational divergence

	

Shaded column indicates the model predicted by the literature and the closest fit to the observed profile

**Table 3:** *Populus* model predictions before (underlying) and after imposition of mutational divergence

	

Shaded column indicates the model predicted by the literature and the closest fit to the observed profile

**Table 4:** *Brassica oleraceae* model predictions before (underlying) and after imposition of mutational divergence

	

In the two shaded columns, the expected (2,3) profile does not fit the observed pattern as well as the (3,3) profile

It can be seen in Table 1 that the profiles of triplet types produced by the different models are very different. If we could observe the triplet profile produced by the branching process underlying a given set of data, we could easily identify which model was responsible. However, we only see the data after mutational processes have applied. When a mutational divergence model is applied to the similarities, a single trajectory of the branching process, producing one ideal type of triplet, can produce many very different data triplets.

We can try to categorize the set of triplets in a set of data by how closely they resemble one of the four basic types. If the two components of the similarity distribution were completely separate, this would also be an easy matter. But the usual large overlap between the components means that we cannot automatically ascribe any data triplet to any particular underlying triplet.

### A statistical approach

As a solution to this problem, we first try to find the best transition, or cutoff point *H* somewhere between the peaks of the two components. For this we compute the product of the probability density at each similarity value less than *H*, according to the component with mean at *t*_1_, and the density at each similarity value greater than *H*, according to the component with mean at *t*_2_, and maximize with respect to *H*. I.e.,

4 $$ {\begin{aligned} H=\max_{h\in(0,1)}\prod_{x\le h}\frac{1}{\sqrt{\sigma_{1}^{2}}}\exp\left[-\frac{(x-\mu_{1})^{2}}{2\sigma_{1}^{2}}\left]\prod_{x> h}\frac{1}{\sqrt{\sigma_{2}^{2}}}\exp\right[-\frac{(x-\mu_{2})^{2}}{2\sigma_{2}^{2}}\right] \end{aligned}}  $$

We then categorize the triplets in the data according to the transition value *H*. If a similarity *x* is less than *H* we classify it as being produced at time *t*_1_, and we write *x*∈*I*. If it is greater than *H*, we classify it as being created at time *t*_2_ and we write *x*∈*J*. This creates eight “octants”, defined by the 8=2^3^ combination of the three triplet similarities, which in turn are collapsed into the four types of triplet in $\mathscr {T}$ tabulated in Table 1, {*t*_1_,*t*_1_,*t*_1_},{*t*_1_,*t*_1_,*t*_2_}(representing three octants), {*t*_1_,*t*_2_,*t*_2_} (representing three octants) and {*t*_2_,*t*_2_,*t*_2_}. The number of triplets of the four types we call the *observed profile*.

Although we can compare the observed profile with the underlying profile, this comparison is not too meaningful since it neglects the fact that many of the data triplets classified as one type may be generated by a different underlying type, not as an error, but simply as a result of the normal process of duplicate gene sequence divergence clearly operative in the more or less dispersed and overlapping components of the distribution of gene pair similarities.

We can, however, take this process into account in producing a *predicted profile* for each model. We first calculate the variance-covariance matrix *Σ* of the *t*_1_ similarities in triplets containing at least of them and *t*_2_ similarities in triplets containing at least two of these. We fixed *covar*(*t*_1_,*t*_2_)=0, in accordance with the Markov nature of the branching process.

For each model $M\in \mathscr {M}$, we construct the *predicted profile* of triplet types by integrating over the trivariate normal with means drawn from the EMMIX analysis or identified by eye with the distribution component peaks, and covariance estimated as above, restricted to the domains defined by the transition point. Thus our prediction of {*t*_1_,*t*_1_,*t*_1_} triplets would involve a restriction to the domain (*I*,*I*,*I*) where all three coordinates are less than or equal to *H*. Our prediction of apparently {*t*_1_,*t*_1_,*t*_2_} triplets would be confined to the three octants where two coordinates are less than or equal to *H* and one is greater. The integrals are weighted by *W*(*Δ*), the expected number of triplets. For example, the predicted number of {*t*_1_,*t*_1_,*t*_2_} triplets would be;

5 $$\begin{array}{@{}rcl@{}} P_{M}&=&\sum_{\Delta\in \mathscr{T}}W_{M}(\Delta)\int_{(I,I,J)\cup(I,J,I)\cup(J,I,I)} N({\mathbf X; \mu,\Sigma})d\mathbf{X}, \end{array} $$

where *μ* is the vector of component means. To summarize, we have defined three types of triplet profile:

- the observed profile, based on triples of genes all having high similarity scores with each other, which can be compiled from the list of gene pairs produced by the SYNMAP function of COGE [1, 2],
- the underlying profile for each model $M\in \mathscr {M}$, produced by the combinatorial probabilities of the branching process for each model $M\in \mathscr {M}$,
- the predicted profile for each model $M\in \mathscr {M}$, produced by grafting a gene pair divergence model on the underlying profile.

For comparative purposes we normalize the underlying and predicted profiles so that the total number of triples is the same as the observed profiles.

## Results

We compare the three profiles for three well-studied flowering plant genomes that are known to have undergone multiple polyploidizations in the last 120 million years, to see if our method predicts the right combination of WGT and WGD.

### Durian

Starting with the durian genome, the (3,3) model, known to represent true evolutionary history, is the only one with a credible prediction profile in Table 2, the only one that has reasonable values for all four triplet types. The three others all fail to predict one or both of the {*t*_1_,*t*_1_,*t*_1_} and {*t*_2_,*t*_2_,*t*_2_} triplets. This indicates the potential of our statistical method, since the original durian sequence article [3] did not recognize the second event as a tripling.

### Poplar

**Fig. 3:**
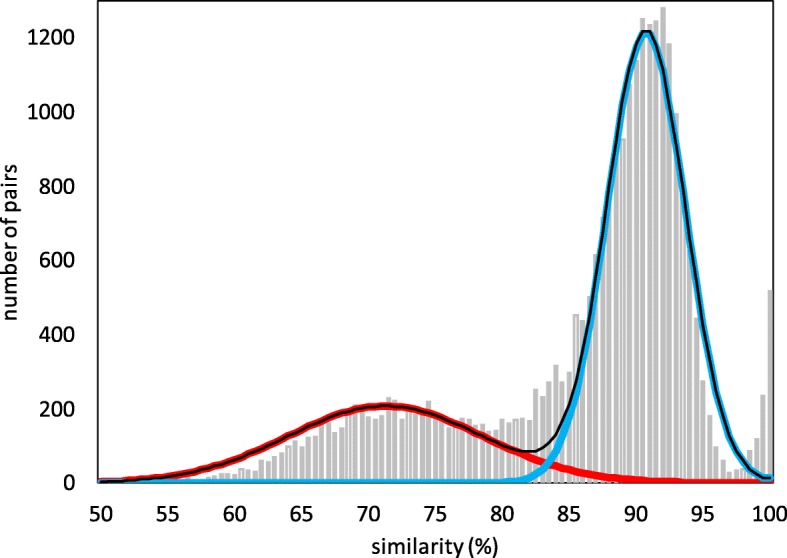
Distribution of syntenic gene pair similarities in *Populus trichocarpa*. Discrimination point *H*=84.5*%*. Cut-off for pairs not originating in polyploidization > 97.5*%*

The predicted profile of the (3,2) model in Table 3 summarizes the true history of the *Populus trichocarpa* genome (COGE ID 25127), whose gene pair similarity distribution is displayed in Fig. 3. Along with *γ*, this shares the ancient “salicoid” WGD with other members of the Salicaceae family [15]. (3,2) is the only model that correctly identifies both the *γ* event as a WGT, and the more recent event as a WGD.

### Cabbage

**Fig. 4:**
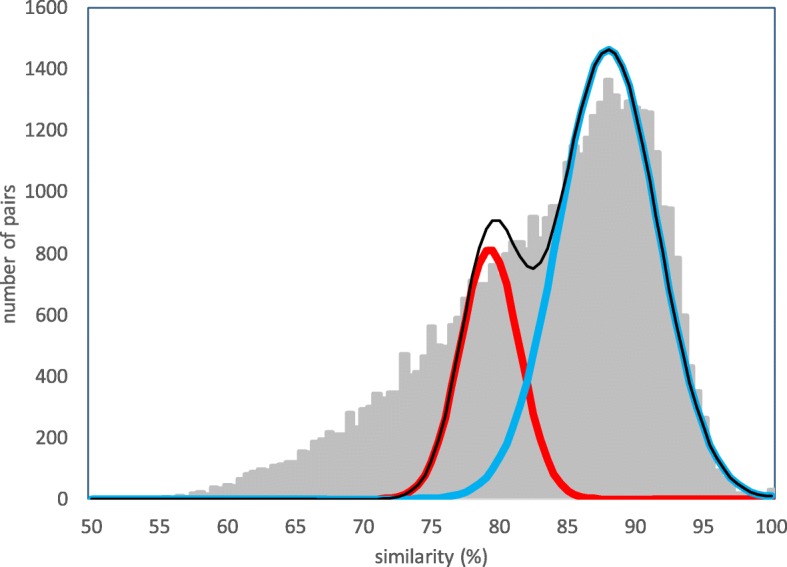
Distribution of syntenic gene pair similarities in *Brassica oleracea*. Discrimination point *H*=83.3*%*. Cut-off for pairs not originating in two recent polyploidization ≤76*%*

The recent ancestor of *Brassica oleracea* genome (COGE ID 26018), underwent a WGT that gave rise not only to the crucifers and mustard genera, but also radishes and other related genera. Early than that a WGD called the *α* doubling is apparent in the whole range of family Brassicacea genera, including *Arabidopsis*. A still earlier WGD, the *β* doubling, occurred in the order Brassicales lineage that includes the Brassicaceae. Thus the cabbage genome counts *γ*,*β*,*α* and a Brassica WGT in its evolutionary history [16, 17]. In Fig. 4, we see that at least the two recent components are clearly distinguishable, so we first carried out an analysis excluding gene pairs of similarity less than 76%.

This analysis in Table 4 does not give satisfactory results. Indeed, the (3,3) profile matches the observed profile much better than the (2,3) profile does. This can be partially attributed to substantial number of similarities generated by the *β* and even *γ* doublings greater than 76%.

We can partially correct this by adding a third event to our branching process. This leads to eight models instead of four, and ten kinds of triplet, summarized in Table 5.

**Table 5:** Formulae for the expected numbers of triplets after three events

Model $M\in \mathscr {M}$			
Triplet $\Delta \in \mathscr {T}$	(2,2,2)	(3,2,2)	(2,3,2)
{*t*1,*t*1,*t*1}	-	*u*^′^(1+*v*)^3^(1+*w*)^3^	-
{*t*1,*t*1,*t*2}	2*uv*(1+*v*)(1+*w*)^3^	2(3*u*^′^+*u*)*v*(1+*v*)(1+*w*)^3^	2*u*(3*v*^′^+*v*)
			×(1+2*v*^′^+*v*)(1+*w*)^3^
{*t*1,*t*2,*t*2}	-	-	-
{*t*2,*t*2,*t*2}	-	-	(1+*u*)*v*^′^(1+*w*)^3^
{*t*2;*t*2;*t*3}	2(1+*u*)*vw*(1+*w*)	2(1+2*u*^′^+*u*)*vw*(1+*w*)	2(1+*u*)(3*v*^′^+*v*)*w*(1+*w*)
{*t*2,*t*3,*t*3}	-	-	-
{*t*3,*t*3,*t*3}	-	-	-
{*t*1,*t*1,*t*3}	2*u*(1+*v*)^2^*w*(1+*w*)	2(3*u*^′^+*u*)(1+*v*)^2^*w*(1+*w*)	2*u*(1+2*v*^′^+*v*)^2^*w*(1+*w*)
{*t*1,*t*3,*t*3}	-	-	-
{*t*1,*t*2,*t*3}	-	-	-
model $M\in \mathscr {M}$			
triplet $\Delta \in \mathscr {T}$	(2,2,3)	(2,3,3)	(3,2,3)
{*t*1,*t*1,*t*1}	-	-	*u*^′^(1+*v*)^3^
			×(1+2*w*^′^+*w*)^3^
{*t*1,*t*1,*t*2}	2*uv*(1+*v*)	2*u*(3*v*^′^+*v*)(1+2*v*^′^+*v*)	2(3*u*^′^+*u*)*v*(1+*v*)
	×(1+2*w*^′^+*w*)^3^	×(1+2*w*^′^+*w*)^3^	×(1+2*w*^′^+*w*)^3^
{*t*1,*t*2,*t*2}	-	-	-
{*t*2,*t*2,*t*2}	-	(1+*u*)*v*^′^	-
		×(1+2*w*^′^+*w*)^3^	
{*t*2;*t*2;*t*3}	2(1+*u*)*v*	2(1+*u*)(3*v*^′^+*v*)	2(1+2*u*^′^+*u*)*v*
	×(3*w*^′^+*w*)(1+2*w*^′^+*w*)	×(3*w*^′^+*w*)(1+2*w*^′^+*w*)	×(3*w*^′^+*w*)(1+2*w*^′^+*w*)
{*t*2,*t*3,*t*3}	-	-	-
{*t*3,*t*3,*t*3}	(1+*u*)(1+*v*)*w*^′^	(1+*u*)(1+2*v*^′^+*v*)*w*^′^	(1+2*u*^′^+*u*)(1+*v*)*w*^′^
{*t*1,*t*1,*t*3}	2*u*(1+*v*)^2^	2*u*(1+2*v*^′^+*v*)^2^	2(3*u*^′^+*u*)(1+*v*)^2^
	×(3*w*^′^+*w*)(1+2*w*^′^+*w*)	×(3*w*^′^+*w*)(1+2*w*^′^+*w*)	×(3*w*^′^+*w*)(1+2*w*^′^+*w*)
{*t*1,*t*3,*t*3}	-	-	-
{*t*1,*t*2,*t*3}	-	-	-
model $M\in \mathscr {M}$			
triplet $\Delta \in \mathscr {T}$	(3,3,2)	(3,3,3)	
{*t*1,*t*1,*t*1}	*u*^′^(1+2*v*^′^+*v*)^3^(1+*w*)^3^	*u*^′^(1+2*v*^′^+*v*)^3^(1+2*w*^′^+*w*)^3^	
{*t*1,*t*1,*t*2}	2(3*u*^′^+*u*)(3*v*^′^+*v*)(1+2*v*^′^+*v*)(1+*w*)^3^	2(3*u*^′^+*u*)(3*v*^′^+*v*)(1+2*v*^′^+*v*)(1+2*w*^′^+*w*)^3^	
{*t*1,*t*2,*t*2}	-	-	
{*t*2,*t*2,*t*2}	(1+2*u*^′^+*u*)*v*^′^(1+*w*)^3^	(1+2*u*^′^+*u*)*v*^′^(1+2*w*^′^+*w*)^3^	
{*t*2;*t*2;*t*3}	2(1+2*u*^′^+*u*)(3*v*^′^+*v*)*w*(1+*w*)	2(1+2*u*^′^+*u*)(3*v*^′^+*v*)(3*w*^′^+*w*)(1+2*w*^′^+*w*)	
{*t*2,*t*3,*t*3}	-	-	
{*t*3,*t*3,*t*3}	-	(1+2*u*^′^+*u*)(1+2*v*^′^+*v*)*w*^′^	
{*t*1,*t*1,*t*3}	2(3*u*^′^+*u*)(1+2*v*^′^+*v*)^2^*w*(1+*w*)	2(3*u*^′^+*u*)(1+2*v*^′^+*v*)^2^(3*w*^′^+*w*)(1+2*w*^′^+*w*)	
{*t*1,*t*3,*t*3}	-	-	
{*t*1,*t*2,*t*3}	-	-	

*u*= probability that two progeny survive after the first polyploidization event. *u*^′^= probability that three survive. Similarly *v* and *v*^′^ are the probabilities that two or three progeny survive, respectively, after the second event. *w* and *w*^′^ are the probabilities that two or three progeny survive after the third event

**Fig. 5:**
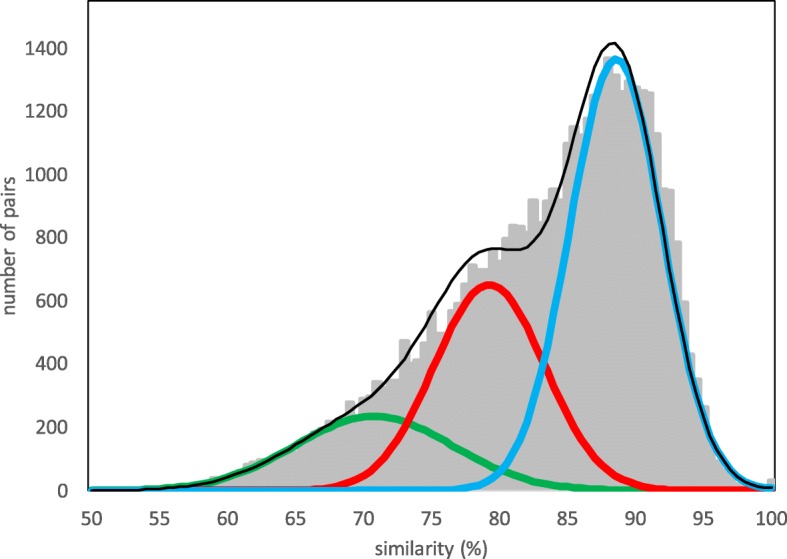
Distribution decomposed into three events. Discrimination points *H*_1_=73*%*,*H*_2_=85*%*

We fix the mean of the first component at 71% to account for the *γ* event, that for the second component, representing the *α* event, at 79.5% and we find two ML discrimination points, as in Fig. 5.

The results of this are shown in Table 6. Here the (3,2,3) model is just as close to the observed profile than the competing (3,3,3) model, with the notable exception of {*t*_2_,*t*_2_,*t*_2_} triplets. The absence of a distinction between the *α* and *β* events means that the similarities they generate are all conflated to yield an excess of *t*_2_, and consequently an excess of *t*_2_ triples, so that a WGT is inferred rather than a WGD.

**Table 6:** *Brassica oleracea* three-event model predictions before (underlying) and after imposition of mutational divergence

	

In the two shaded columns, the expected (3,2,3) profile fits the observed pattern as well as the (3,3,3) profile

The obvious remedy for this would be to construct four-event models (sixteen of them), with profiles consisting of 20 different triplets. We leave this for further work. In general the number of models is exponential: 2^*m*^ for *m* events, while the number of triples follows the polynomial (i.e., cubic) tetrahedral sequence (A00292 in [18]) $\frac {1}{6}m(m+1)(m+2)$, so that eventually there would not be enough data to discriminate among the models. Choosing among models with different numbers of events would require some standard for model selection such as the Akaike or Bayesian information criteria.

## Conclusions

We model the process of fractionation to account for the distribution of gene pair similarities after a number of whole genome doublings, triplings, etc., each followed by a period of duplicate gene loss. The model is a discrete-time branching process, with synchronous birth number *r*_*i*_≥2 across the *i*−*th* generation population and deaths determined by a *r*_*i*_-nomial law conditioned on at least one survivor.

The observations of gene pair similarities consist of a mixture of normals, each component generated by one event, with the event time estimated by the sequence divergence from the event to the present. Despite the overlapping distributions, we can estimate the mean (*via* a local mode), standard deviation and proportion of the sample.

Statistics on gene pairs alone do not allow us to infer *r*_*i*_, so we introduce the study of gene triplets. We find formulae for the expected number of each kind of triplet, categorized as to which events produced the similarities among the three pairs of genes.

We develop a way of grafting a gene divergence model on this underlying profile of triplets to produce a predicted profile of the number of triplets of each kind. This can then be compared with the observed number of triplets.

### Further work

Distinguishing among the four models combining tetraploidization or hexaploidization in two successive events is the simplest example of a more general problem. The theoretical way is clear to extending these ideas to include, for example, octoploidization through the extraction of quadruplets instead of triplets. In addition, there is a straight forward extension to the case of three or more successive polyploidization events, which we have undertaken in the study of *Brassica oleracea*. Here, three events, three normal components and two transition points are estimated from the distribution of similarities. The combinatorial probabilities have been worked out for this case and many others, and the methodology is available to complete this.

It is true that in some cases, such as that we presented in [9], concerning *Durio zibethinus*, the ploidy is evident from the clear presence in the SYNMAP self-comparison dotplots of sets of *r* regions covering a large proportion of the genome, each set represented by exactly $\binom {r}{2}$ synteny blocks showing synteny among all *r* regions. (*r*=3 in the case of *Durio*.) Clear cases like this are rare, however, especially for genomes where the last polyploidization is more remote in time.

In previous work [7], we used additional information, beyond that contained in the similarity distribution, to confirm the recent hexaploidization of *Brassica rapa* against the alternative of tetraploidization. This kind of data, however, namely speculation about the number of single-copy genes in the current genome, was extremely subjective in that report, and is unreliable even when assessed by the best available methods on well-assembled and annotated genomes.

A distribution of gene pair similarities is generated in the comparison of two related genomes as well as in the self-comparison of a single genome. The number of orthologous gene pairs available when comparing two related genomes is generally much greater than the number of paralogous pairs identified in the self-comparison of two genomes, simply because the loss by fractionation of one copy of a duplicated gene does not eliminate all related orthology pairs: the other remaining copy and its orthology pairs remain intact. This suggests an avenue to improved accuracy of polyploidy levels inference. The larger number of data, however, may not always compensate for the fact that the speciation component of the similarity distribution is always the most recent one [9], so that the more remote (earlier) components associated with polyploidy become statistically less clear and informative.

## Data Availability

The datasets analysed during the current study are available in the COGE repository [1, 2], https://genomevolution.org/coge/

## Abbreviations

ML: Maximum likelihood
WGD: Whole genome doubling
WGT: Whole genome tripling
